# The relationship between systemic inflammation index, systemic immune-inflammatory index, and inflammatory prognostic index and 90-day outcomes in acute ischemic stroke patients treated with intravenous thrombolysis

**DOI:** 10.1186/s12974-023-02890-y

**Published:** 2023-09-30

**Authors:** Fei Ma, Lulu Li, Liang Xu, Jiacheng Wu, Aimei Zhang, Junqi Liao, Jingyi Chen, Yunze Li, Li Li, Zhaoyao Chen, Wenlei Li, Qing Zhu, Yuan Zhu, Minghua Wu

**Affiliations:** 1 Department of Neurology, Affiliated Hospital of Nanjing University of Chinese Medicine, Jiangsu Province Hospital of Chinese Medicine, 155 Hanzhong Road, Nanjing, 210029 China; 2 Friend Plastic Hospital of Nanjing Medical University, Nanjing, 210029 China

**Keywords:** Systemic inflammatory index, Systemic immune-inflammatory index, Inflammatory prognostic index, Acute ischemic stroke, Intravenous thrombolysis

## Abstract

**Background and purpose:**

To explore the association of systemic inflammatory index (SIRI), systemic immune-inflammatory index (SII) and inflammatory prognosis index (IPI) with 90d outcomes in patients with acute ischemic stroke (AIS) after intravenous thrombolysis.

**Methods:**

The patients who underwent intravenous thrombolysis were enrolled in the present study from September 2019 to December 2022. According to the relevant blood indexes obtained in 24 h after admission, the corresponding values of SIRI, SII and IPI were calculated. The correlation among SIRI, SII, IPI, and admission NIHSS scores was examined by Spearman correlation analysis. ROC curve analysis was conducted to determine the optimal cut-off value of SIRI, SII, IPI, and their corresponding sensitivity and specificity to evaluate their predictive value on admission for poor prognosis. To investigate whether high SIRI, SII, and IPI were independent predictors of poor outcomes within 90 days, variables with *P*-value < 0.05 during univariate analysis were included in multivariate analysis.

**Results:**

Compared with the good outcome group, the poor outcome group had higher SIRI, IPI, and SII. Spearman correlation analysis showed that the SIRI, IPI, and SII levels significantly correlated with the admission NIHSS score (*r* = 0.338, 0.356, 0.427, respectively; *P*_s_ < 0.001). Univariate analysis and Multivariate logistic regression analysis revealed high SIRI, SII, and IPI values as independent risk factors for poor 90-day prognosis (OR = 1.09, 1.003 and 7.109, respectively).

**Conclusions:**

High SIRI, IPI, and SII values are correlated with poor 90d outcomes in AIS patients undergoing intravenous thrombolysis.

## Introduction

Approximately 70% of all strokes are cases of acute ischemic stroke (AIS), which results from blockage or a clot that impedes blood vessels carrying blood and oxygen to the brain [1]. This condition is known for its high mortality and disability rates, causing a significant burden on families and society [2]. Intravenous thrombolytic therapy has become the mainstay of treatment for AIS within a 4.5-h time window. Our previous studies found that NLR was associated with the prognosis, recurrence, and death of acute ischemic stroke at 90 days [3]. Herein, we further analyzed the correlation of NLR-based composite inflammatory indicators with the prognosis of AIS patients that underwent intravenous thrombolysis.

In recent years, three novel inflammatory markers have been introduced. We explored the association of systemic inflammatory index (SIRI), systemic immune-inflammatory index (SII), and inflammatory prognosis index (IPI) with 90d outcomes in AIS patients undergoing intravenous thrombolysis by analyzing the correlation with NLR-based composite inflammatory indicators.

SIRI, IPI, and SII represent novel predictive indices derived from the neutrophil-to-lymphocyte ratio (NLR), combining various inflammatory parameters. These composite ratios offer a practical and reproducible approach to predicting prognosis. Moreover, they can be integrated with other biomarkers to develop more robust prediction models, facilitating more precise and timely decision-making in clinical practice. Although some studies have shown that SIRI, IPI and SII can be used as a potential and valuable tool to predict the short-term prognosis of stroke patients, there are few clinical reports, and the study population may have limitations caused by ethnic differences [4]. Therefore, we made a detailed analysis of the relevant cases in local area, in order to explore the relationship between SIRI, IPI, SII and short-term prognosis of patients with hyperacute venous thrombolysis in acute cerebral infarction, in order to provide a predictive tool for precision treatment [5].

## Materials and methods

### Study design and participants

We used data from the stroke center of Jiangsu Province Hospital of Chinese Medicine from September 2019 to December 2022. An observational cohort study of 232 patients with acute ischemic stroke who underwent intravenous thrombolysis with alteplase (rt-PA) was enrolled. The study included 190 patients with acute ischemic stroke who met the following inclusion criteria: (1) aged over 18 years old; (2) diagnosed with AIS according to the Chinese AIS diagnosis and treatment guidelines; (3) received rt-PA intravenous thrombolysis within 4.5 h of symptom onset [6]; (4) premorbid mRS ≤ 2. Exclusion criteria consisted of (1) bridging therapy patients; (2) patients with malignant tumors, autoimmune diseases, or blood system diseases; (3) acute or chronic infections; (4) severe hepatic or renal insufficiency; (5) patients with missing follow-up data.

The Institutional Research Review Board of the Affiliated Hospital of the Nanjing University of Chinese Medicine approved this study which was conducted in accordance with the Declaration of Helsinki (2017NL-012-01).

### Definition of inflammatory indicators

The systemic inflammatory index (SIRI) which uses peripheral blood monocytes and the neutrophil-to-lymphocyte ratio (NLR); the systemic immune-inflammatory index (SII), which incorporates platelets and the neutrophil-to-lymphocyte ratio (NLR), and the inflammatory prognosis index (IPI), which takes into account C-reactive protein (CRP), neutrophil-to-lymphocyte ratio (NLR), and serum albumin protein (ALB) as a prognostic index.

### Data acquisition

Upon admission, all participants underwent a standard assessment of their demographic characteristics, including sex, age, National Institutes of Health Stroke Scale (NIHSS) score, Modified Rankin Scale (mRS) scale, vascular risk factors (hypertension, diabetes, atrial fibrillation, coronary heart disease, prior stroke, smoking, and alcohol history), and preonset medication (antiplatelet drugs, statins, and anticoagulants).

Within 24 h of admission, all AIS patients underwent assessment using the NIHSS, which ranged from 0 to 42. Patients were classified as mild (NIHSS ≤ 5) and moderate-to-severe (NIHSS > 5) stroke [7]. The modified Rankin score (mRS) was used to assess the 90-day outcomes during follow-up, with a score of 0–2 indicating a favorable prognosis and a score of 3–6 indicating a poor prognosis [8].

### Measurement of composite inflammatory ratios based on blood tests

Samples of venous blood were taken within 24 h of admission. If blood tests were conducted more than once during the 24-h period, we used the results of the first test. We recorded the data of neutrophils (N), lymphocytes (L), platelets (PLT), monocytes (M), albumin (ALB), and high-sensitivity C-reactive protein (Hs-CRP). These counts were used to determine the inflammatory biomarkers listed below: neutrophil-to-lymphocyte ratio (NLR = N/L), systemic immune-inflammation index (SII = P × [N/L]), system inflammation response index (SIRI = N × [M/L]) and inflammatory prognosis index (IPI = CRP × NLR/ALB).

### Statistical analysis

The Mann–Whitney or T-tests were used to compare continuous factors based on the data distribution, whereas Chi-square tests were used to compare categorical data. The Spearman correlation test examined the association between each indicator and the NIHSS score. Each indicator's ability to discriminate for a 90-day prognosis was tested using the receiver operating characteristic (ROC) curve, and the optimal cut-off value was identified based on the greatest sum of sensitivity and specificity. Risk variables with a *P*-value of less than 0.05 during univariate analysis were incorporated into the multivariate analysis. The results were presented as odds ratios (ORs) and 95% confidence intervals (CIs). Kaplan–Meier survival curves were plotted based on the optimal cut-off value, and log-rank tests were conducted for each indicator. All statistical analyses were performed using the Statistical Package for the Social Sciences 25.0 (SPSS; IBM, USA), and a two-sided *P* < 0.05 was statistically significant.

## Results

**Fig. 1:**
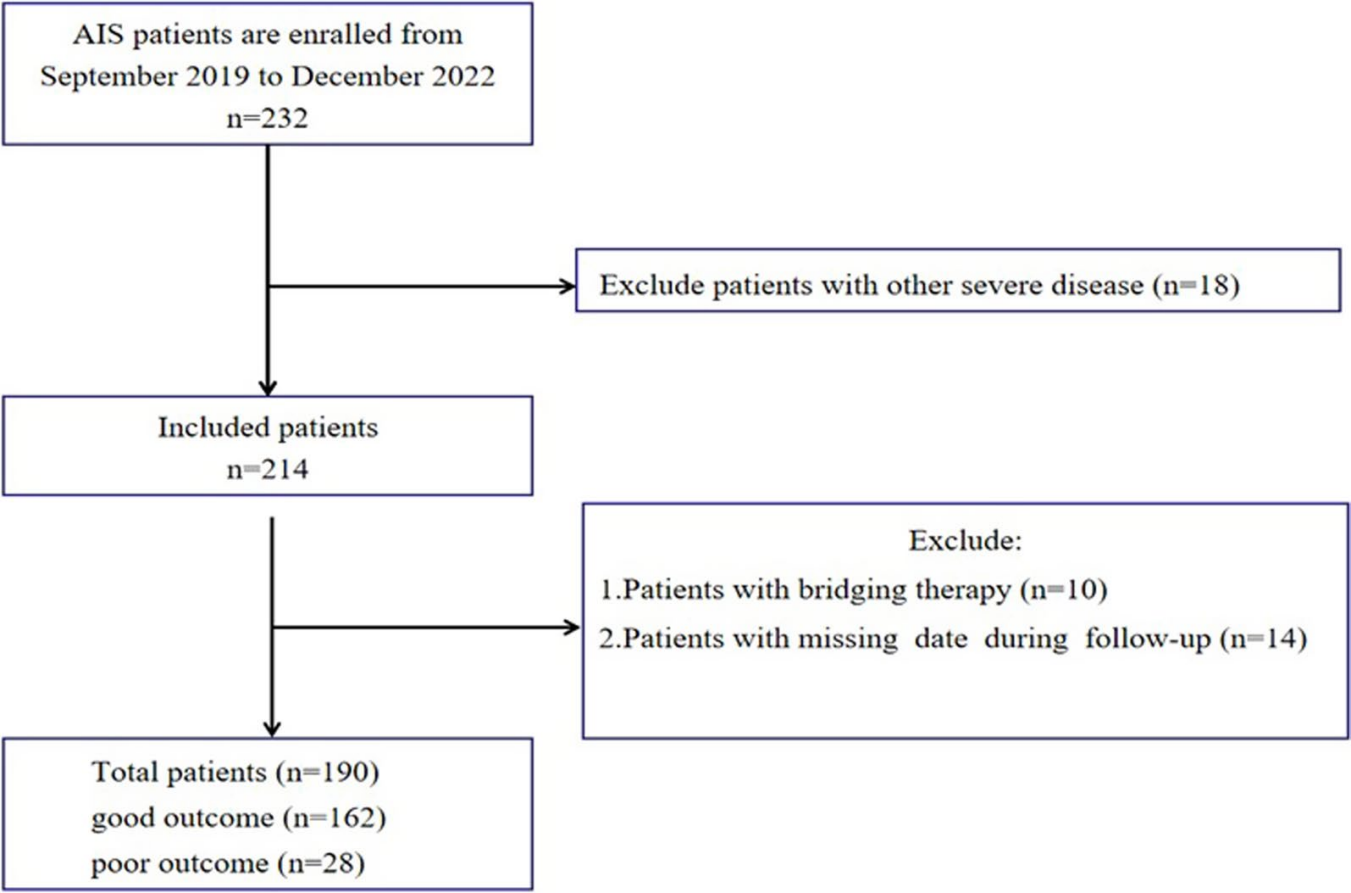
Flowchart of patient selection. CHD: coronary heart disease; AF: atrial fibrillation; NIHSS: National Institutes of Health Stroke Scale; MPV: mean platelet volume; NLR: neutrophil-to-lymphocyte ratio; CRP: C-reactive protein; IPI: inflammatory prognosis index; SII: systemic immune-inflammation index; SIRI: system inflammation response index; RBG: random blood glucose

232 AIS patients that underwent intravenous thrombolysis were screened in this study. A total of 42 patients were excluded, due to severe liver and kidney dysfunction, and metastasized tumors (*n* = 18), bridging treatments after intravenous thrombolysis (*n* = 10), and incomplete follow-up data (*n* = 14). Ultimately, a total of 190 subjects were included for the final analysis. A flowchart showing the patient selection is shown in Fig. 1.

### Patient characteristics

Baseline characteristics of the study participants are provided in Table 1. Compared to the good prognosis group, the poor prognosis group had higher age, baseline NIHSS scores, neutrophil counts, premorbid mRS scores, and Random Blood Glucose (RBG), NLR, SIRI, IPI, and SII values but lower lymphocyte counts.

**Table 1:** Demographics and clinical characteristics of AIS patients with different prognoses

Characteristics	Total (*n* = 190)	Good prognosis group (*n* = 157)	Poor prognosis group (*n* = 33)	*P*
Gender (male, *n*, %)	122 (64.2)	102 (65.0)	20 (60.6)	0.635
Age, mean (± SD)	70.389 ± 11.675	69.854 ± 11.201	72.939 ± 13.416	0.100
Vascular risk factors				
Smoking, *n* (%)				
Yes	51 (26.8)	45 (28.7)	6 (18.2)	0.217
No	139 (73.2)	112 (71.3)	27 (81.8)	
Drinking, *n* (%)				
Yes	28 (14.7)	26 (16.6)	2 (6.1)	0.122
No	162 (85.3)	131 (83.4)	31 (93.9)	
Diabetes, *n* (%)				
Yes	56 (29.5)	46 (29.3)	10 (30.3)	0.908
No	134 (70.5)	111 (70.7)	23 (69.7)	
Hypertension, *n* (%)				
Yes	128 (67.4)	104 (66.2)	24 (72.7)	0.470
No	62 (32.6)	53 (33.8)	9 (27.3)	
CHD, *n* (%)				
Yes	33 (17.4)	25 (15.9)	8 (24.2)	0.252
No	157 (82.6)	132 (84.1)	25 (75.8)	
AF, *n* (%)				
Yes	13 (6.8)	11 (7.0)	2 (6.1)	0.845
No	177 (93.2)	146 (93.0)	31 (93.9)	
*Medical history*				
Antiplatelet, *n* (%)				
Yes	44 (23.2)	37 (23.6)	7 (21.2)	0.771
No	146 (76.8)	120 (76.4)	26 (78.8)	
Statins, *n* (%)				
Yes	27 (14.2)	22 (14.0)	5 (15.2)	0.865
No	163 (85.8)	135 (86.0)	28 (84.8)	
Antihypertensive, *n* (%)				
Yes	117 (61.6)	95 (60.5)	22 (66.7)	0.509
No	73 (38.4)	62 (39.5)	11 (33.3)	
Hypoglycemic drug, *n* (%)				
Yes	52 (27.4)	42 (26.8)	10 (30.3)	0.677
No	138 (72.6)	115 (73.2)	23 (69.7)	
TOAST, *n* (%)				
LAA	110 (57.9)	92 (58.6)	18 (54.5)	0.029
CE	25 (13.2)	16 (10.2)	9 (27.3)	
SAO	47 (24.7)	43 (27.4)	4 (12.1)	
Unclassified	8 (4.2)	6 (3.8)	2 (6.1)	
OCSP, *n* (%)				
TACI	34 (17.9)	25 (15.9)	9 (27.3)	0.142
PACI	63 (33.2)	52 (33.1)	11 (33.3)	
LACI	69 (36.3)	62 (39.5)	7 (21.2)	
POCI	24 (12.6)	18 (11.5)	6 (18.2)	
Premorbid mRS, *n *(%)				
0	55 (28.9)	46 (29.3)	9 (27.3)	< 0.001
1	105 (55.3)	86 (54.8)	19 (57.6)	
2	30 (15.8)	25 (15.9)	5 (15.2)	
NIHSS, median [IQR]	4.000 [3.000,6.000]	3.000 [2.000,5.000]	7.000 [6.000,10.000]	< 0.001
SBP, mean (± SD)	153.000 ± 23.806	152.204 ± 23.473	156.788 ± 24.983	0.317
DBP, mean (± SD)	85.637 ± 14.868	84.701 ± 14.799	90.091 ± 14.381	0.056
*Laboratory data*				
LDL, median[IQR]	2.750 [2.130,3.320]	2.750 [2.130,3.320]	2.810 [2.140,3.280]	0.875
HDL, median [IQR]	1.260 [1.080,1.500]	1.260 [1.100,1.500]	1.220 [1.060,1.390]	0.458
RBG, median [IQR]	6.180 [4.920,8.650]	6.050 [4.910,7.680]	7.420 [5.820,10.570]	0.024
Lymphocyte, median [IQR]	1.750 [1.260,2.350]	1.830 [1.330,2.410]	1.450 [0.930,1.770]	< 0.001
Monocyte, median [IQR]	0.500 [0.390,0.660]	0.510 [0.390,0.660]	0.460 [0.400,0.670]	0.946
Neutrophil, median [IQR]	4.420 [3.290,5.550]	4.320 [3.210,5.210]	4.970 [4.130,8.250]	0.001
Platelet, median [IQR]	186.000 [153.000,224.000]	188.000 [154.000,224.000]	177.000 [153.000,209.000]	0.372
MPV, median [IQR]	10.300 [9.700,11.200]	10.300 [9.700,11.200]	10.400 [9.700,11.200]	0.818
NLR, median [IQR]	2.368 [1.596,3.893]	2.159 [1.463,3.566]	3.509 [2.838,7.237]	< 0.001
CRP, median [IQR]	0.500 [0.500,1.740]	0.500 [0.500,1.500]	0.740 [0.500,4.260]	0.107
Albumin, median [IQR]	40.600 [37.700,42.900]	40.400 [37.700,42.600]	41.600 [37.700,43.500]	0.176
IPI, median [IQR]	0.047 [0.025,0.136]	0.043 [0.023,0.113]	0.109 [0.041,0.522]	< 0.001
SII, median [IQR]	451.644 [293.248,715.759]	413.571 [264.923,654.741]	636.842 [461.159,1088.124]	< 0.001
SIRI, median [IQR]	1.179 [0.732,2.036]	1.083 [0.707,1.844]	1.912 [1.298,3.726]	< 0.001

### Correlation between IPI, SII, and SIRI level and admission NIHSS score in different groups of AIS patients

**Fig. 2:**
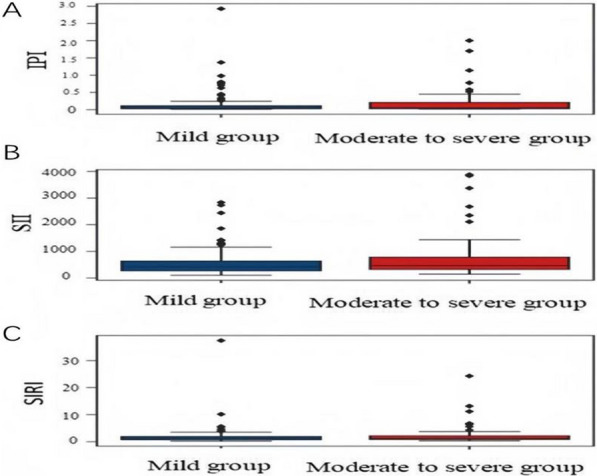
The differences between IPI, SII, and SIRI level and admission NIHSS Score. **A** Differences in IPI levels between two groups of AIS patients. **B** Differences in SII levels between two groups of AIS patients. **C** Differences in SIRI levels between two groups of AIS patients. IPI: inflammatory prognosis index; SII: systemic immune-inflammation index; SIRI: system inflammation response index; NIHSS: National Institutes of Health Stroke Scale

Spearman correlation analysis revealed a significant correlation between IPI, SII, and SIRI values with NIHSS score (*r* = 0.338, 0.356, and 0.427, respectively, *P* < 0.05). IPI, SII, and SIRI levels in moderate-to-severe AIS patients (NIHSS score ≥ 5) remained higher than in mild AIS patients (NIHSS score < 5) (median: 0.101 vs. 0.034, 675.669 vs. 377.752, 1.685 vs. 1.010), according to the Mann–Whitney *U*-test (Fig. 2A–C).

### Levels of composite inflammatory indicators with different prognosis

**Fig. 3:**
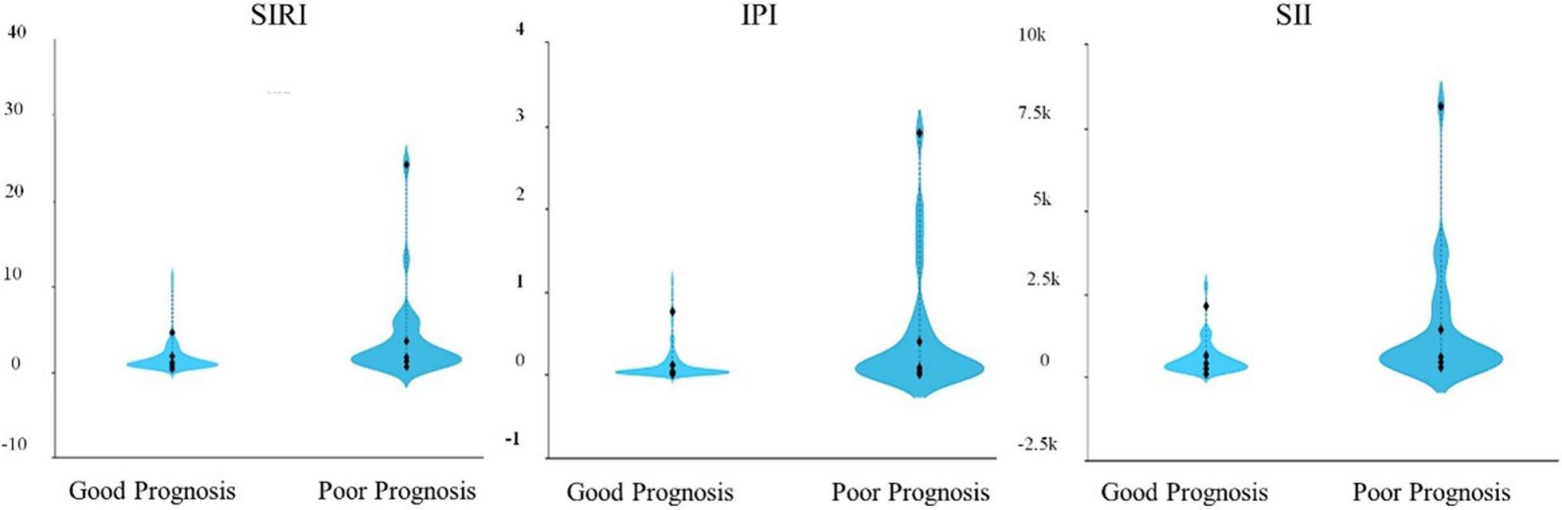
The violin plot of composite inflammatory ratios. The violin plot of the distribution of SIRI, IPI, and SII among different prognosis groups (*P* < 0.05). IPI: inflammatory prognosis index; SII: systemic immune-inflammation index; SIRI: system inflammation response index; NIHSS: National Institutes of Health Stroke Scale

Figure 3 shows the violin plots of SIRI, IPI, and SII between the two groups. Patients in the good prognosis group had lower SIRI, IPI, and SII levels than in the poor prognosis group (*P* < 0.05).

### Prognostic value of SIRI, IPI, and SII in AIS patients

**Fig. 4:**
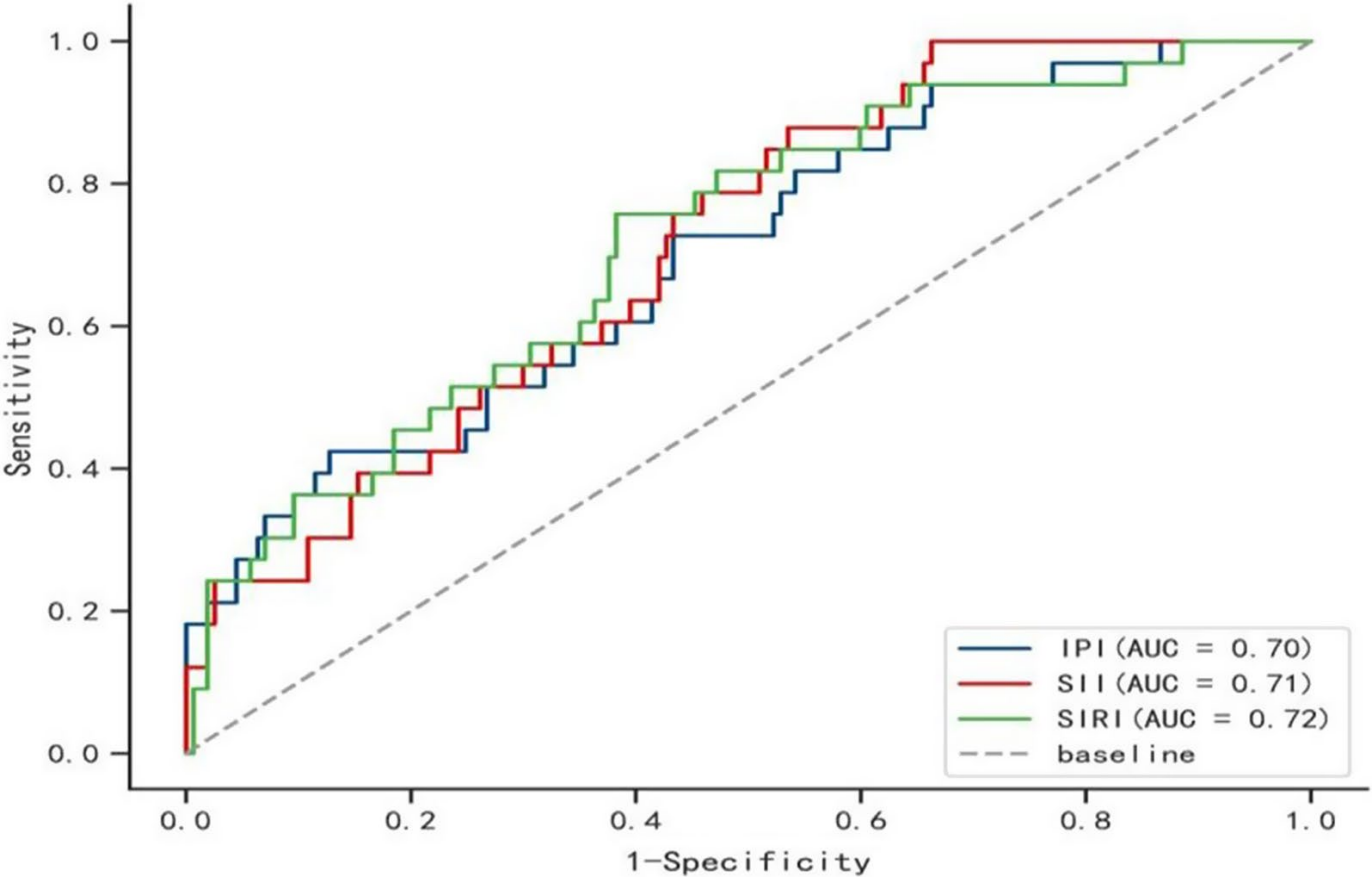
The ROC value of SIRI, IPI, and SII in predicting poor short-term outcomes in AIS patients. The AUC of IPI was 0.701 with a sensitivity of 0.424 and a specificity of 0.873; the AUC of SII was 0.715 with a sensitivity of 0.879 and a specificity of 0.465; the AUC of SIRI was 0.720 with a sensitivity of 0.758 and a specificity of 0.618. IPI: inflammatory prognosis index; SII: systemic immune-inflammation index; SIRI: system inflammation response index

ROC analysis revealed that a SIRI cut-off value of 1.298 × 10^9^ /L demonstrated a sensitivity of 0.758 and a specificity of 0.618 for poor 3-month outcome, with an AUC of 0.720 (95% CI 0.612–0.751, *P* < 0.05) (Fig. 4). The SII index's predictive accuracy was poor. A 3-month poor outcome was differentiated by the SII cut-off value of 392.903 × 10^9^ /L, which had a sensitivity of 0.879, a specificity of 0.465, and an AUC of 0.715 (95% CI 0.546–0.826; *P* < 0.05). An IPI cut-off value of 0.223 yielded a sensitivity of 0.424 and specificity of 0.873 for a poor 3-month outcome, with an AUC of 0.701 (95% CI 0.604–0.826, *P* < 0.05).

### Univariate and multivariate logistic analysis of SIRI, SII, IPI, and poor 3-month outcome in AIS patients

During univariate regression analysis, the admission NIHSS scores, premorbid mRS, NLR, SIRI, SII, and IPI were significantly associated with poor outcomes at 3 months (*P* < 0.05). To ascertain whether elevated SIRI, SII, and IPI were independent prognostic markers of poor outcomes within 3 months, variables with *P* < 0.05 during the binary analysis were included in multivariate analysis.

The presence of comorbidities were considered as potential risk factors for AIS. These factors were included as confounding variables, despite not showing a statistically significant difference between age, smoking, alcohol history and prognosis. However, based on multivariate logistic regression analysis, it was found that high SIRI, SII, and IPI values were independently associated with poor prognosis at 3 months (OR = 1.091, 1.003, 7.109; *P* < 0.05) (Table 2).

**Table 2:** Univariate and multivariate logistic analysis of SIRI, SII, IPI, and poor 3-month outcome in AIS patients

	Model 1		Model 2		Model 3	
	OR (95% CI)	*P*-value	OR (95% CI)	*P*-value	OR (95% CI)	*P*-value
SIRI	1.122 (1.018,1.236)	0.021	1.123 (1.017,1.240)	0.022	1.091 (1.002,1.187)	0.045
SII	1.004 (1.002,1.006)	< 0.001	1.004 (1.002,1.006)	< 0.001	1.003 (1.001,1.005)	0.013
IPI	7.566 (2.144,26.702)	0.002	8.590 (2.239,32.953)	0.002	7.109 (1.659,30.462)	0.008

Model 1 was univariate analysis. SIRI, SII, and IPI were associated with different outcomes at three months (*P* < 0.05)

Model 2 was adjusted for gender and age

Model 3 was adjusted for gender, age, random blood glucose, neutrophil, lymphocyte, admission NIHSS scores, smoking, drinking history, premorbid mRS, TOAST and comorbidities. High SIRI, SII, and IPI (*P* < 0.05) were independent predictors of poor 3-month prognosis, according to multivariate logistic regression analysis (OR: odds ratio; CI: confidence interval)

### Kaplan–Meier survival curve of each indicator

**Fig. 5:**
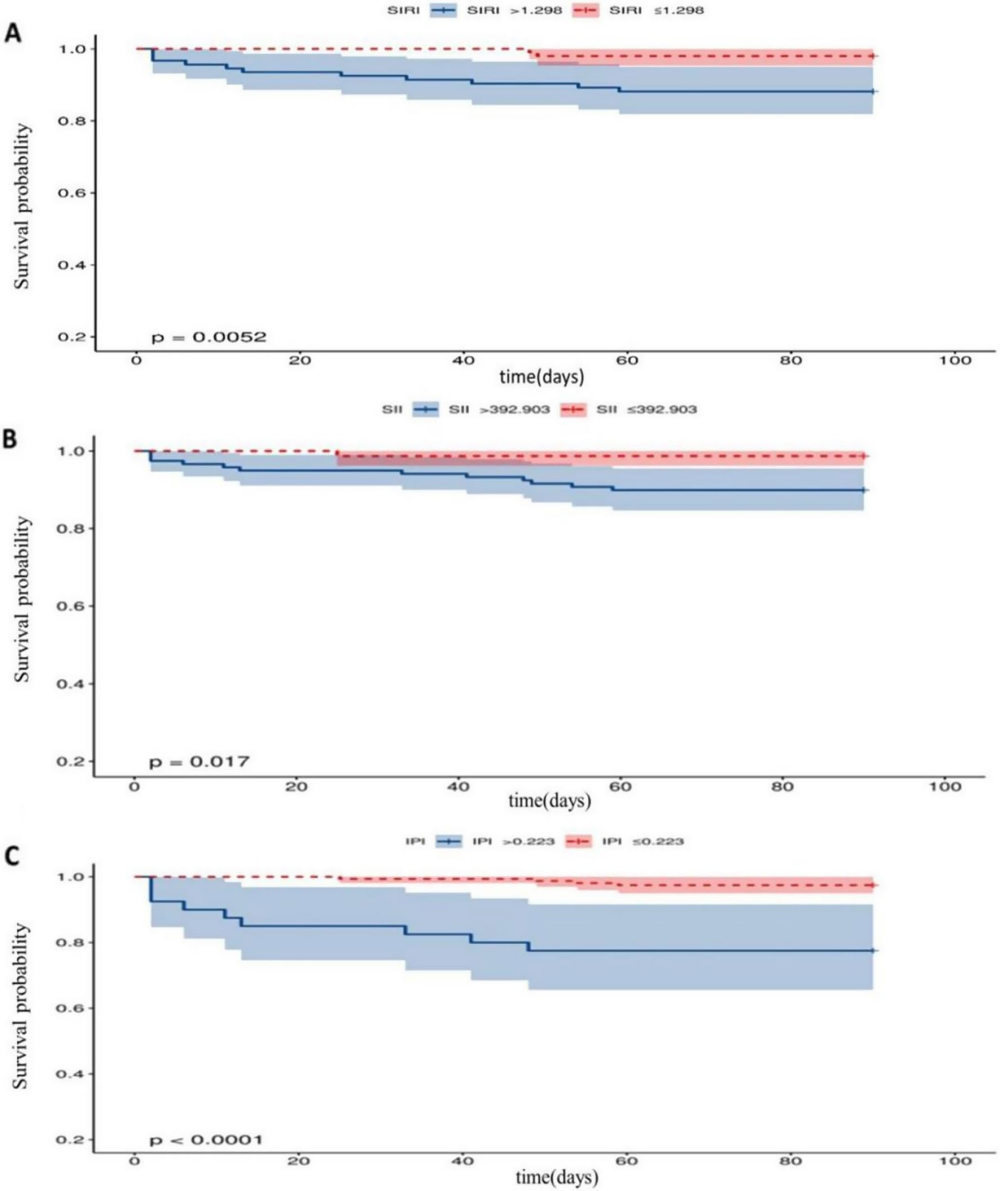
Kaplan–Meier survival curve for 90-day outcome in patients with AIS. **A** Kaplan–Meier survival curve for 90-day mortality in AIS patients according to SIRI; **B** Kaplan–Meier survival curve for 90-day mortality in AIS patients according to SII; **C** Kaplan–Meier survival curve for 90-day mortality in AIS patients according to IPI. Kaplan–Meier survival curve showed that SIRI > 1.298, SII > 392.903, and IPI > 0.223 were associated with a low risk of 90-day recurrence or mortality (*P* < 0.05). IPI: inflammatory prognosis index; SII: systemic immune-inflammation index; SIRI: system inflammation response index

This study also investigated whether each indicator was associated with 90-day recurrence or mortality. The information on recurrence or mortality is given in Table 3. As shown in Fig. 5, SIRI > 1.298 × 10^9^, SII > 392.903 × 10^9^, and IPI > 0.223 were associated with the risk of 90-day recurrence or mortality.

**Table 3:** The information on recurrence or mortality

	Total (*n* = 190)	Recurrence (*n* = 6)	Mortality (*n* = 7)
SIRI > 1.298 × 10^9^	87 (45.8%)	5 (5.7%)	6 (6.9%)
SIRI ≤ 1.298 × 10^9^	103 (54.2%)	1 (1.0%)	1 (1.0%)
SII > 392.903 × 10^9^	113 (59.5%)	5 (4.4%)	7 (6.2%)
SII ≤ 392.903 × 10^9^	77 (40.5%)	1 (1.3%)	0 (0%)
IPI > 0.223	34 (17.9%)	2 (5.9%)	7 (20.6%)
IPI ≤ 0.223	156 (82.1%)	4 (2.6%)	0 (0%)

## Discussion

SIRI, IPI, and SII are linked to the advancement of cardiovascular and neoplastic illnesses, according to several clinical studies [9–11]. The platelet–neutrophil interaction contributes to clinical ischemia illness and micro thrombosis in stroke, according to several human clinical trials and animal studies [12–14]. Neutrophils have been linked to stroke severity, infarct size, and prognosis in studies [17]. Although the neutrophil-to-lymphocyte ratio has been associated with the prognosis of AIS, research on composite inflammatory markers such SIRI, IPI, and SII is lacking. The link between SIRI, IPI and SII and the short-term prognosis of intravenous thrombolysis in AIS patients was further investigated in our study. We discovered that SIRI, IPI, and SII performed well in predicting AIS patients' 90-day prognosis. A higher risk of death or recurrence was linked to higher SIRI, IPI, and SII levels. Additionally, SIRI, IPI, and SII were independently linked with short-term prognosis in AIS patients, according to results from univariate and multivariate binary logistic regression.

There have been many studies on the inflammatory reaction in the acute stage of cerebral infarction. In eMCA rats, the circulating neutrophil count increased after tPA administration significantly more than in the control group, according to Shi et al. [32]; at an early stage, there is a noticeable buildup of neutrophils and T cells in the microvascular lumen of the ipsilateral cerebral hemisphere. At the same time, it was discovered that in patients, the population of neutrophils and T cells in peripheral blood rose quickly after tPA infusion [33], indicating that tPA may have worsened the patient's internal environment's imbalance of homeostasis before it led to further issues. The study [34] also discovered that lymphocyte–endothelial cell interactions encourage microvascular dysfunction and inflammatory factor production, which result in neuronal cell death and disruption of the blood–brain barrier, and that blocking lymphocyte and neutrophil transport can lessen intracerebral hemorrhage brought on by tPA. Therefore, it is reasonable to think that intravenous thrombolysis is involved in the progression of inflammation while undergoing therapeutic treatment.

It is not clear how intravenous thrombolysis leads to the increase of SIRI, IPI and SII and short-term poor prognosis in patients with acute cerebral infarction. Here are some potential mechanisms: stroke occurrence can result in significant damage of synaptic glial cells, astrocytes, and neurons. The blood–brain barrier is damaged and brain edema is exacerbated during AIS, where neutrophils are first produced in the infarction core and penumbra [15]. These neutrophils release inflammatory factors that cause damage to the endothelial cell membrane and basement membrane [16]. Microglia are activated and circulating leukocytes penetrate periinfarct or infarct foci in the acute phase of ischemic stroke, according to preclinical and clinical proof-of-concept studies. After a stroke, resident and invading cells collaborate to control the inflammatory response [35]. It has been proved that a significant number of inflammatory chemicals are released during an acute ischemic stroke [36].

For SIRI, which was employed to investigate the part that monocytes play in AIS using NLR. Monocytes have early migration and infiltration in AIS. They differentiate into macrophages under the influence of OX-LDL, ingest low-density lipoprotein to produce foam cells, and subsequently release a variety of inflammatory substances [18]. By secreting VEGF, activated monocytes also make blood vessels more permeable and break down the blood–brain barrier [19]. Our findings confirmed that the SIRI index performs better in representing the status of systemic inflammatory response and is an independent predictor of prognosis than peripheral blood cell ratios like NLR, LMR, and PLR. Poor 90-day prognosis in AIS patients was independently predicted by a high SIRI index. We found that for AIS patients with SIRI > 1.298 × 10^9^, the incidence of poor prognosis, including the risk of recurrence or death, was increased at 90 days.

According to the available data, IPI is correlated with CRP, NLR, and ALB (IPI = CRP × NLR/ALB). The primary protein in human blood, albumin, regulates colloid osmotic pressure and body nutrition [21]. Numerous physiological processes carried out by albumin include thrombus formation, platelet aggregation inhibition, anti-oxidation, and anti-inflammation [22]. Albumin has been demonstrated to have neuroprotective benefits in investigations on AIS animal models, and clinical research have confirmed that low albumin levels might negatively impact AIS prognosis [23, 24]. The degree of inflammation in the body during the beginning of an infection is clinically reflected by the CRP index. The severity, infarct size, and prognosis of AIS patients have all been linked to CRP levels [25]. The vascular endothelium, among other cells, may interact with CRP to induce vascular inflammation, unstable or even rupture atherosclerotic plaque, and subsequently generate thrombi [26, 27]. A growing body of evidence points to a link between elevated CRP levels and the risk of cardiovascular and cerebrovascular events, as well as death from any cause [28–30]. A meta-analysis consistently demonstrated an association between CRP levels and the probability of death in AIS [31].

For SII, it has been determined that the condition replicates three critical pathophysiological processes in AIS: thrombosis, inflammation, and immune response. A high SII is indicative of thrombogenic and immunological dysregulation states, both of which have been linked to serious adverse outcomes [20]. Our findings demonstrate that the best SII threshold (392.903 × 10^9^) performs well in predicting the prognosis of AIS patients at 90 days. Its accuracy is, however, less than that of the SIRI and IPI index.

There are several limitations in this study that should be acknowledged. It is crucial to remember that this was a retrospective analysis based on stroke registration data rather than a multi-center clinical trial. Future research should think about doing multi-center prospective studies as it presents a possible bias and reduces the generalizability of the findings. The analysis did not include any patients who had mechanical thrombectomy, which may have limited the data' application to this particular subgroup. Finally, it needs to be understood that composite inflammatory indices might change a lot while a patient is in the hospital. Instead of relying exclusively on measurements taken prior to thrombolysis or at admission, future research should concentrate on dynamically monitoring these indices throughout the whole treatment process.

Our findings corroborate that higher SIRI, SII, and IPI values indicate greater disease severity at admission and an increased incidence of poor outcomes at 90-day prognosis. As a result, these indices offer a new approach to conducting stratified interventions in AIS patients, potentially leading to improved prognosis in clinical practice.

## Conclusion

To be honest, we are the first NLR-based comprehensive analysis of SIRI, SII, and IPI and the short-term prognosis of intravenous thrombolysis therapy for acute cerebral infarction, and the inflammatory indicators after stroke are significantly related to the short-term prognosis. SIRI, SII and IPI, which are easily accessible blood indicators without subjective bias, will provide theoretical support for early intervention in patients with acute cerebral infarction.

## Data Availability

The data that support the findings of this study are available from the corresponding author upon reasonable request.

## Abbreviations

AIS: Acute ischemic stroke
NLR: Neutrophil-to-lymphocyte ratio
PLR: Platelet-to-lymphocyte ratio
LMR: Lymphocyte-to-monocyte ratio
HT: Hemorrhagic transformation
OTT: Onset to treatment time
PAO: Proximal arterial occlusion
TOAST: Trial of Org 10172 in Acute Stroke Treatment
TC: Total cholesterol
TG: Triglyceride
HDL: High-density lipoprotein
LDL: Low-density lipoprotein
FBG: Fasting blood glucose
Hs-CRP: Hypersensitive C-reactive protein
OR: Odds ratio
95% CI: 95% Confidence interval
AUC: Area under the curve
